# Retraction: Plumbagin Suppresses the Invasion of HER2-Overexpressing Breast Cancer Cells through Inhibition of IKKα-Mediated NF-κB Activation

**DOI:** 10.1371/journal.pone.0207273

**Published:** 2019-01-18

**Authors:** The PLOS ONE Editors

After publication of this article [1], concerns were raised about the data reported in Figs 2A and 3B. Several areas within these figures include cell staining patterns that appear to be repeated within and across figure panels, and in some areas the background pixelation appears highly similar across panels. Furthermore, some of the areas are highly similar to staining patterns reported by another group, in Fig 1 of [2]. A member of *PLOS ONE*’s Editorial Board confirmed that the invasion assay results reported in these figures are critical in supporting the conclusions of the *PLOS ONE* article.

In discussing these concerns with the authors and a representative at the Gdański Uniwersytet Medyczny, the authors stated that Figs 2 and 3, as well as some Western blots and accompanying densitometry data, were generated by a third-party company whose involvement in this research was not declared in the published article. The company confirmed that they had provided invasion assay and Western blot data for this article, including data presented in Figs 2A and 3B. The company provided images in support of some results in question, but the concerns about image similarities were not fully resolved based on our examination of these data, and some of the original data have not been provided.

The authors provided replication data supporting the results in question but were not able to clarify the concerns raised about the published figures.

In light of the concerns about the integrity of data reported in Figs 2 and 3, and the absence of raw data supporting the published results, the *PLOS ONE* editors retract this article.

AK and AD did not agree with retraction.
